# Case of Vesico-Vaginal Fistula

**Published:** 1874-10

**Authors:** J. E. O’Brien

**Affiliations:** Scranton, Pa.


ARTICLE II.—*A Case of Vesico-Vaginal Fistula.* By J. E. O'BRIEN, M.D., Scranton, Pa.

Mrs. O'H——, of Dunmore, Pa. began to suffer from incontinence of urine two weeks subsequent to a severe and protracted labor. On examination I found the cause to be a rent in the bladder, situated about the centre of the vesico-vaginal septum, and of a size sufficient to admit readily the index finger. Of course every drop of urine dribbled through it, producing the usual symptoms, which always render life miserable to these sufferers. I waited a month for the patient to get well over the effects of parturition, and on July 14th made the operation, as follows: One assistant (Dr. Fisher) held the speculum, one administered anæsthetics, and a third attended to sponges. Having pared the edges of the fistule, I introduced six silver sutures, and then considered whether I should use Sims' or Bozeman's method of fastening them. Being prepared to use either method, I chose the former, for the following reasons, which I have taken pains to analyze and arrange as a contribution to the study of this subject:

1. Sims' method is simpler.

2. It reveals to sight and touch the condition of each stitch, and permits full control of its tension, so that after twisting all the wires the surgeon can tighten (or loosen) some which will probably require such adjustment.

3. It permits inspection and treatment of the wound throughout the after treatment of the case.

4. It permits the consistency of the cicatrix to be tested by sight and touch before removal of the stitches, and after removal of each stitch; so that

5. Alternate stitches may be removed, or one stitch a day, which may sometimes be a prudent precaution, as it is in harelip and in cleft palate.

The removal of one stitch may reveal the necessity for retaining the others, which can be done in Sims' method.

On the other hand, Bozeman's method is

1. A little more difficult, keeping the patient longer under ether; it is too nice a point to require each hole in the leaden shield to correspond exactly with each wire.

2. It hides the condition of the stitches, even before they are tightened, and places a leaden shield between their tension and our knowledge of it.

3. It hides the wound from sight and treatment. (It is said also to protect it from the secretions, but I think syringing quite as effectual, especially if facilitated by the use of Sims' speculum.) As to supporting the wound, which the shield is said to do, there would seem to be some doubt of its utility, while there can be no doubt that the suspension of a piece of lead by the stitches increases their tension.

4. It hides the consistency of the cicatrix from sight and touch, until control of it is partially resigned.

5. It compels disturbance of all the stitches in removing any of them.

I admit that all these points of difference are slight, but they may be considered sufficient to turn the scale of preference until larger statistics declare more positively on the subject.

As between the methods of Sims and Bozeman, I find by reference to a few authorities at hand, that Gross declares in favor of Sims' method, except, perhaps, in vesico-uterine and urethro-vaginal cases; so does Emmet. Thomas is non-commital, praising both. Hamilton seems to prefer Bozeman's method. Gardner, in a note to his translation of Scanzoni, expresses decided preference for Sims'.

In conclusion, it is only fair to say that Dr. Bozeman deserves the very greatest credit for his great success in treating every form of fistule. In looking over his monograph I am deeply impressed with the importance of his labors in vesico-uterine and other complications.

It may be interesting here to note the success of Simon, of Heidelberg (see Thomas' work) with silk sutures, which success would undoubtedly be greater with silver wire, especially in large fistulæ.

The shields or splints of Simpson, Baker Brown, Agnew, Baltey, *et al.*, are no doubt inferior to Dr. Bozeman's.

My patient was kept constipated for two weeks. Finding the cicatrix firm, I removed all the sutures on the eighth day, and removed the catheter on the twelfth, when the patient urinated *per vias naturales.* She got up on the fourteenth day, and remains perfectly cured.

She passed some sand through the fistula before the operation, and the catheter was almost filled with phosphatic deposit afterwards, but this symptom gave no further trouble.

This operation requires less light than is needed for incision of the cervix uteri, which I have made after Sims' method.

There is a slight defect in most of the tenaculi made for uterine surgery; they are bent at nearly a right angle, which should be more acute to prevent slipping.

HISTORY.

"For centuries, steady, persevering and systematic efforts have been made to render this revolting malady curable."—(Thomas.) "Velpeau, in 1839, thus speaks of cure of these fistulæ: 'To abrade the borders of an opening, when we do not know where to grasp them; to shut it up by means of needles or thread, when we have no point apparently to secure them; to act upon a movable partition placed between two cavities, hidden from our sight, and upon which we can scarcely find any purchase, seems to be calculated to have no other result than to cause unnecessary suffering to the patient.' Vidal de Cassis says: 'I do not believe that there exists in the science of surgery a well authenticated, complete cure of vesico-vaginal fistula.'"—(Thomas.)

There were, no doubt, occasional cures in exceptional and fortunate cases from the time when Ambrose Pare described the disease in the sixteenth century until our day; yet Druitt's Surgery (1848) dismisses this operation in two lines, and recommends a rubber bag to catch the urine. The elements of success may have been discovered, but were not yet combined and utilized.

Later still (1861), Gardner, referring to his translation of Scanzoni, says: "In the foregoing dissertation upon urinary fistulæ, the author evidently shows that in his opinion (and he represents German, if not European knowledge,) there is little hope for the poor woman suffering from a severe form of this disease; and the statistics of European surgery—little reliable as some of them are—support this view. We say 'little reliable,' for many of these surgeons claim success where it does not exist. The operations of these great surgeons are public; the *results* private."

Dr. Valentine Mott stated in public (Report of First Anniversary of the N. Y. Woman's Hospital, 1856): "I was present when eight cases were operated upon—seven by Jobert and one by

Roux, two of the most distinguished surgeons in Europe—*and all of them failed.*"

Honor, then, to American genius, which has by the labors of Marion Sims especially, and of Bozeman, Emmet and others, combined the essentials to success in this operation, and which has made cure the rule instead of the exception.

I know of no case in which the transition from misery to health and comfort is more marked than in the patient who is cured of vesico-vaginal fistula.

